# Dissemination of a Web-Based Tool for Supporting Health Insurance Plan Decisions (Show Me Health Plans): Cross-Sectional Observational Study

**DOI:** 10.2196/jmir.9829

**Published:** 2018-06-20

**Authors:** Jingsong Zhao, Nageen Mir, Nicole Ackermann, Kimberly A Kaphingst, Mary C Politi

**Affiliations:** ^1^ Huntsman Cancer Institute Salt Lake City, UT United States; ^2^ Division of Public Health Sciences Department of Surgery Washington University in Saint Louis St. Louis, MO United States; ^3^ Department of Communication University of Utah Salt Lake City, UT United States

**Keywords:** Affordable Care Act, health insurance decision aid, health literacy, public health

## Abstract

**Background:**

The rate of uninsured people has decreased dramatically since the Affordable Care Act was passed. To make an informed decision, consumers need assistance to understand the advantages and disadvantages of health insurance plans. The Show Me Health Plans Web-based decision support tool was developed to improve the quality of health insurance selection. In response to the promising effectiveness of Show Me Health Plans in a randomized controlled trial (RCT) and the growing need for Web-based health insurance decision support, the study team used expert recommendations for dissemination and implementation, engaged external stakeholders, and made the Show Me Health Plans tool available to the public.

**Objective:**

The purpose of this study was to implement the public dissemination of the Show Me Health Plans tool in the state of Missouri and to evaluate its impact compared to the RCT.

**Methods:**

This study used a cross-sectional observational design. Dissemination phase users were compared with users in the RCT study across the same outcome measures. Time spent using the Show Me Health Plans tool, knowledge, importance rating of 9 health insurance features, and intended plan choice match with algorithm predictions were examined.

**Results:**

During the dissemination phase (November 2016 to January 2017), 10,180 individuals visited the SMHP website, and the 1069 users who stayed on the tool for more than one second were included in our analyses. Dissemination phase users were more likely to live outside St. Louis City or County (*P*<.001), were less likely to be below the federal poverty level (*P*<.001), and had a higher income (*P*=.03). Overall, Show Me Health Plans users from St. Louis City or County spent more time on the Show Me Health Plans tool than those from other Missouri counties (*P*=.04); this association was not observed in the RCT. Total time spent on the tool was not correlated with knowledge scores, which were associated with lower poverty levels (*P*=.009). The users from the RCT phase were more likely to select an insurance plan that matched the tool’s recommendations (*P*<.001) compared with the dissemination phase users.

**Conclusions:**

The study suggests that a higher income population may be more likely to seek information and online help when making a health insurance plan decision. We found that Show Me Health Plans users in the dissemination phase were more selective in the information they reviewed. This study illustrates one way of disseminating and implementing an empirically tested Web-based decision aid tool. Distributing Web-based tools is feasible and may attract a large number of potential users, educate them on basic health insurance information, and make recommendations based on personal information and preference. However, using Web-based tools may differ according to the demographics of the general public compared to research study participants.

## Introduction

A key strategic goal of the Affordable Care Act (ACA) is to extend affordable coverage to the uninsured [1]. Since the enactment of the ACA, 20 million consumers have gained health insurance coverage and the uninsured rate dropped to a historic low of 8.6% [2,3]. In addition, the ACA improved access to primary care and medications, decreased mortality, and overall improved health outcomes among vulnerable populations [4].

Although the ACA led to better access to care for many, some consumers, especially those new to health insurance, had difficulty understanding health insurance details and using selected health insurance plans [5,6]. Making well-informed decisions about health insurance in the ACA marketplace requires individuals to understand the complex benefits and trade-offs of each insurance plan option and compare them to select the best choice for them. Recognizing a need for better consumer support, many national and state-wide organizations drafted recommendations that organizations should adopt to help consumers identify effective plans [7-9]. In addition, marketplace enrollment assistance programs were created to support consumer choices in-person, by telephone, and through outreach events in most states.

Despite public education and outreach efforts, health insurance literacy remains a critical barrier impacting enrollment decisions [10]. Limited knowledge about health insurance may hinder consumers’ abilities to select a suitable health plan and use it to obtain health care. Many in-person assistance programs have very large caseloads, and are not able to reach everyone who might need guidance selecting insurance [11]. Given the budget cuts in the 2018 enrollment cycle [12], providing comprehensive in-person assistance has become even more challenging.

Web-based resources may be an effective way to supplement in-person assistance for learning about health insurance and plan selection. Web-based support may be particularly important for reaching disadvantaged populations with limited access to in-person outreach, or those who require more guidance than can be accomplished in face-to-face meetings; online tools may promote health equity among these groups [13,14]. However, developing and disseminating Web-based resources are different from in-person support workshops and may require systematic dissemination and implementation plans to reach target populations.

In order to distribute information on federal health plans and assist consumers with health insurance plan selection in the ACA marketplace, we created the Show Me Health Plans (SMHP) Web-based decision support tool. Designed for those with limited health insurance literacy, SMHP delivered health insurance education and calculated estimated annual costs for each user based on Medical Expenditure Panel Survey (MEPS) data for their age, gender, and self-reported health conditions. It then sorted health insurance plans in the ACA marketplace by lowest to highest annual cost, and provided ranges of potential annual costs across the marketplace. The effectiveness of the SMHP tool was examined in a randomized controlled trial (RCT), and described in previous publications [15,16]. Study participants who used SMHP had higher health insurance knowledge, decision self-efficacy, confidence in their health plan choice, and improved health insurance literacy compared to participants using the federal health insurance exchange website [15,16]. They were also more likely to select plans that better matched their health care needs.

After the RCT, in response to the promising effectiveness of SMHP in a research context and the growing need for Web-based health insurance decision support, the study team released the SMHP tool so that it was available to the general public. We used principles of dissemination and implementation science to guide efforts in translation and adoption of research evidence to the target population throughout the state of Missouri. There is increasing interest and investment in disseminating and translating effective study interventions more broadly to target populations [17-19]. Political support, funding agency priorities, capacity of the dissemination organization, and researchers’ knowledge on applying study findings can influence the success of study translation [20-23]. Although many researchers have dissemination expectations from funders [20], the majority of public health professionals spend little time and effort on program dissemination [24], and few studies reported sustainability and challenges of disseminating an online decision aid tool from an effective intervention study. To address this gap, we examined the use of the SMHP tool during its dissemination to the general public in Missouri and compared this with use of the tool during the RCT.

## Methods

### Overview

This study used a cross-sectional observational design to examine the public dissemination of the SMHP tool across Missouri. Dissemination phase users were compared with users from a RCT study previously carried out by the authors. In brief, the RCT recruited English-speaking participants, aged 18 to 64 years, not eligible for Medicaid, and living within 90 miles of St. Louis, Missouri. Enrolled participants (n=328) were randomly allocated to the SMHP intervention group or to the HealthCare.gov control group [15,16]. In the dissemination phase, the SMHP tool had information on page one about users who might benefit most from the SMHP tool (ie, people living in Missouri eligible for the ACA marketplace), but anyone could access the information without a login ID or access criteria. On the first page of the tool, visitors were notified that some of the information they entered would be used for scientific research, but all of their information was anonymous and not connected to identifying information.

**Table 1:** Key expert recommendations for implementing evidence-based interventions.

Recommendation	Strategies	Implementation examples
Develop stakeholder interrelationships	Identify and prepare champions Build a coalition Identify and prepare champions Use advisory boards and workgroups	The study team built up coalitions with local health nonprofit organizations, community action agencies, and local health care centers and departments throughout the randomized controlled trial and dissemination phases These community partners were regularly updated on study findings, received updated website information for their use, and continued to communicate with the study team about health insurance reform and decision support
Train and educate stakeholders	Conduct educational meetings Conduct educational outreach visits Develop educational materials Inform local opinion leaders	Several strategies were utilized to educate stakeholders, including developing educational materials, conducting educational meetings and outreach visits, and informing local opinion leaders
Use evaluative and iterative strategies	Assess for readiness and identify barriers and facilitators Conduct local needs assessment	Local needs assessments were conducted to collect information on the SMHP^a^ website The study team assessed the likelihood of adoption and implementation of the website, along with potential barriers and facilitators to implementation
Adapt and tailor to context	Promote adaptability Tailor strategies	Website changes made to the tool adapted based on collected feedback
Engage consumers	Use mass media	SMHP was featured on local television news, shared via social media, and shared via electronic newsletters to reach large number of consumers and health policy experts

^a^SMHP: Show Me Health Plans.

### Dissemination of the SMHP Tool

The study team relied on several expert recommendations for implementation of evidence-based interventions when disseminating the SMHP tool [25,26]. Key strategies are summarized and displayed in Table 1.

### Tool Content Changes

The organization of the SMHP tool during the RCT phase included five sections: (1) *Welcome* (to introduce the goals of the tool); (2) *Let’s Learn* (to educate users on different topics important to know prior to purchasing health insurance coverage); (3) *Let’s Review* (to measure the user’s knowledge/understanding of key terms); (4) *Eligibility* (to assess whether or not the user is eligible for Marketplace plans based on the information provided); and (5) *Your Plans* (to display good-fit plans based on the information the user inputs in the eligibility section). In the trial, users had to view all sections in order. During the dissemination phase, users could reach sections in any order except the last section. This allowed SMHP users to skip sections and choose the ones that they wanted to view.

Four types of changes were made between the RCT and dissemination phases based on stakeholder feedback, including design changes, content changes, page section changes, and wording changes. Design changes (eg, darken text, increase size of image, label a *Next* button rather than simply display an arrow) were to help SMHP users navigate through the tool effectively. Content changes included reiterating statements on preventive care, pre-existing conditions, and out-of-pocket maximum; adding a new pregnancy question to better calculate Medicaid eligibility; updating the list of health conditions assessed to generate a more precise cost estimate of health care expenses for each user; and adding a link pointing to additional information resources. Content changes were made based on new ACA policies and suggestions from stakeholders and community members. A *Simple Choice Plan* page was added based on new policy changes in the marketplace, and a *Gateway to Better Health* program page was added to inform those in St. Louis City and County about a bridge program to provide limited coverage to those who were ineligible for Medicaid since Missouri did not expand the program. Furthermore, RCT study information was deleted and wording changes were made to add clarity to the tool content.

### Measures

For this analysis, demographic information, including age, gender, income, federal poverty level, number of chronic conditions, and county of residence were collected. During the analysis, users in the dissemination phase were divided into two groups: those who started using the tool but did not finish all the sections (ie, started group), while the finished tool group was defined as those who finished the tool and saw their health insurance plan options.

#### Use of the Tool

Each time a user logged on to the tool, a unique session ID was generated, which enabled each visit to be tracked. Each time a page on the tool was accessed, a tracking database stored the session ID, date and time, as well as user actions (ie, whether they logged in, logged out, viewed the page, or redirected). Session IDs were randomly generated and created so that users could not be identified.

#### Knowledge

Knowledge was measured using eight questions in the *Let’s Review* section. The scale was developed based on our past work assessing health insurance knowledge [15,16]. Knowledge was assessed based on the percentage of people answering each item correctly.

#### Importance Rating

In the *Let’s Review* section, users were asked to rate the importance of nine insurance features from least to most important on a scale of 1 (not at all important) to 5 (very important). The nine features were: cost of health insurance premium, cost of deductible, cost of doctor visits, cost of prescription pills or medicine, choice of doctors (including some that are out-of-network), cost of out-of-network care, fixed costs for tests or care, out-of-pocket maximum, and formulary.

#### Match With Preferences and Algorithm Predictions

In the *Your Plans* section, the tool recommended three good-fit insurance plans based on participant’s eligibility and estimated costs across available plans. It also invited users to select an intended plan choice from the entire list of available plan options. Match with preferences was assessed by comparing the participant’s intended plan choice with the most important features. Matches were categorized as good, moderate, or poor, using methodology described in prior papers [15,16]. Good matches included plan selections that included features participants rated as important to them. For example, if participants rated premiums as “very important” (ie, a rating of 4 or 5), and ranked premium cost as most important to their plan choice, and chose a plan with a premium in the lowest 25% out of plans available, it was considered a good match. Moderate matches would include plans with some features rated as important to participants (eg, rating premium as “very important” and most important to plan choice, then selecting a plan with a premium in the lowest 50% of all plans). Poor matches were plans that did not include many features participants rated or ranked as important. Match with algorithmic predictions was calculated by comparing how many SMHP users selected one of the plans that was displayed as a “good fit” plan based on their demographics and health care needs.

### Data Analysis

Descriptive statistics were calculated for demographics, time spent using the tool, importance ratings, and plan choice match stratified by phase of tool use. Means and standard deviations or frequencies and percentages are presented for all variables. Additionally, range is presented for time spent using the tool, and medians and interquartile ranges are presented for importance rankings. We conducted bivariate analyses to test for associations between phase of tool use (RCT versus dissemination phase) and demographics; between those who began the eligibility portion and those who completed the eligibility portion to view plans; time spent using the tool, importance ratings, and plan choice match. Chi-square tests were used for categorical variables, and *t*-tests were used for parametric continuous variables, using a Satterthwaite adjustment for inequality of variances when appropriate, or a Wilcoxon rank sum test or Kruskal-Wallis test for nonparametric data. For correlations obtained for time usage between RCT and dissemination phases across demographics and knowledge scores, Pearson’s correlation was obtained for parametric data, and Spearman’s correlation was obtained in instances where data were nonparametric. SAS version 9.4 was used for analyses.

## Results

### Participant Characteristics

During the dissemination phase (November 9, 2016 to January 31, 2017), 10,180 individuals visited the SMHP tool. Of those 10,180 individuals, 1069 stayed on the tool for at least one second, suggesting that they did not exit after briefly viewing the home page. The mean age of SMHP users (n=386), who began the eligibility section in the dissemination phase, was 43.6 years (SD 14.4), more than half were female (212/374, 56.7%), 52% came from St. Louis City or County (196/374), and 57% had one or more chronic conditions (201/350, see Table 2). Comparing the dissemination phase users to the RCT users, the first group was more likely to live outside St. Louis City or County (*P*<.001), were less likely to be below the federal poverty level (FPL; *P*<.001), and had a higher income (mean US $40,523 versus US $30,407, *P*=.03).

We compared the characteristics of users who began the eligibility section of the tool (n=56) to those who completed the eligibility section and saw plans (n=330) for the dissemination phase. No significant differences were found between the 2 groups in terms of age, gender, county of residence, and FPL, but those who finished the tool had higher income (mean US $41,085 versus US $14,000, Wilcoxon Rank Sum |Z| approximation=2.8, *P*=.005), and were significantly more likely to have a chronic condition (59.1% versus 30.0%; χ^2^=6.5, *P*=.01).

### Use of the Show Me Health Plans (SMHP) Tool

The median total time for tool usage for the 1069 dissemination phase users was 0.9 minutes (range 0.02-189.1; Table 3). Three-quarters of participants spent 7.5 minutes or less on the tool. One hundred and thirty SMHP users viewed each page of the tool’s five sections with a median time of 17 minutes (range 3.5-189.1). This was significantly lower than time spent by RCT users (*P*=.001), who had a median time of 21.5 minutes (range 6.3-175.1). Compared to the RCT phase, users spent less time on the *Welcome* (*P*<.001), *Let’s Learn* (*P*<.001), *Let’s Review* (*P*<.001), *Eligibility* (*P*<.001), and *Your Plans* (*P*<.001) sections. All users in the dissemination phase started on the *Welcome* section (1069/1069, 100%) and of those users, 46% (488/1069) went on to view the *Let’s Learn* section, 34% (362/1069) went on to view the *Let’s Review* section, 43% (459/1069) went on to view the *Eligibility* section, and 31% (331/1069) went on to view the *Your Plans* section. Users in the RCT phase were required to go through the entire tool so their usage did not differ.

**Table 2:** Demographics of users from the dissemination and randomized controlled trial (RCT) phases.

Variable		Dissemination phase		RCT phase	Test statistic		*P* value	
Age^a^ (n=386), mean (SD)		43.6 (14.4)		43.1 (13.2)	–0.4^b^		.69	
**Gender (n= 374), n (%)**								
	Male		162 (43.3)	67 (40.9)		0.28^c^		.59
	Female		212 (56.7)	97 (59.1)				
**County (n=374), n (%)**								
	St. Louis City or County		196 (52.4)	152 (92.7)		81.0^c^		<.0001
	Other		178 (47.6)	12 (7.3)				
Income^d^ (n= 337), mean (SD)		40,523.20 (38,867)		30,407.01 (54,402)	–2.13^b^		.03	
**Federal poverty level (n=334), n (%)**								
	<100%		45 (13.5)	72 (43.9)		63.6^c^		<.0001
	100%-249%		152 (45.5)	64 (39.0)				
	250%-399%		79 (23.7)	15 (9.2)				
	≥		58 (17.4)	13 (7.9)				
**Number of chronic conditions (n= 350), n (%)**								
	0		149 (42.6)	68 (41.5)		0.06^c^		.81
	≥1		201 (57.4)	96 (58.5)				

^a^n=386 (dissemination phase); n=164 (RCT).

^b^Refers to *t* values.

^c^Refers to χ^2^ values.

^d^n=337 (dissemination phase); n=164 (RCT). All values in US $.

**Table 3:** Time spent using the Show Me Health Plans (SMHP) tool by users in the dissemination and the randomized controlled trial (RCT) phases.

Variable		Dissemination phase^a^			RCT phase			Wilcoxon rank sum, |Z| approximation	*P* value^c^
		n	Median time in sec (IQR^b^)	Range (sec)	n	Median time in sec (IQR)	Range (sec)		
**Section**									
	*Welcome*	1069	7.0 (20.0)	1-1751	164	19.0 (22.5)	5-260	8.78	<.001
	*Let’s Learn*	488	75.5 (228.5)	1-4967	164	284.5 (245.0)	26-2328	9.67	<.001
	*Let’s Review*	362	116.0 (217.0)	1-3081	164	383.5 (217.5)	144-1685	14.38	<.001
	*Eligibility*	459	132 (167.0)	1-4085	164	279.0 (171.5)	94-1013	10.19	<.001
	*Your Plans*	331	138 (324)	1-8319	164	180.5 (292.5)	18-7662	4.07	<.001
All included^d^		1069	0.9 (7.5)	0.02-189.1	164	21.5 (15.1)	6.3-175.1	17.0	<.001
Completed entire tool^d^		130	17.0 (17.8)	3.5-189.1	164	21.5 (15.1)	6.3-175.1	3.24	.0012
At least started each section^d^		229	12.2 (15.6)	1.0-189.1	164	21.5 (15.1)	6.3-175.1	7.27	<.001

^a^Only users with greater than zero seconds time are included in time calculations (ie, those who just clicked are not included).

^b^IQR: interquartile range.

^c^Testing difference in time between dissemination and RCT phases.

^d^Values refer to overall time; times are expressed in minutes.

SMHP users from St. Louis City or County spent more time overall on the tool than those from other Missouri counties in the dissemination phase (Wilcoxon Rank Sum |Z| approximation=2.01, *P*=.04); this association was not observed in the RCT group. Age (*r*=.24, *P*<.001) was positively correlated to overall time spent using SMHP in both phases; however, it had a stronger positive correlation with overall time spent using SMHP in the RCT phase (*r*=.32, *P*<.001). The number of chronic conditions was only positively associated with overall time spent using SMHP in the RCT phase (*r*=.23, *P*=.003).

### Knowledge Scores

In the dissemination phase, SMHP users had a mean knowledge score of 89.5% (SD 15.3), compared to 77.4% (SD 18.2) for users in the RCT. The total time spent on the tool was not correlated with knowledge scores. In both phases, the knowledge score was directly associated with the percentage of FPL (Kruskal-Wallis χ^2^=9.0, *P*=.03 [RCT] and Kruskal-Wallis χ^2^ =11.5, *P*=.009 [dissemination phase]).

### Importance Ranking

Out of nine categories, cost of health insurance premium (126/182, 69.2%) and choice of doctors (110/171, 64.3%) were ranked as the most important factors when considering a health insurance plan in the dissemination phase, while costs of out-of-network care (29/173, 16.9%) received the highest percentage of not important rankings. In contrast, out-of-pocket maximum (111/164, 67.7%) and cost of health insurance premium (108/164, 65.9%) received the highest percentage of most important rankings in the RCT, while choice of doctors (8/164, 4.9%) and cost of out-of-network care (7/164, 4.3%) received the highest percentages of not important rankings. The mean importance rankings of cost of doctors’ visits (*P*=.002), cost of prescription pills or medicine (*P=*.002), cost of doctors (*P=*.004), cost of out-of-network care (*P*<.001), fixed cost for tests or care (*P*<.001) and out-of-pocket maximum (*P*=.01) were different in the dissemination phase and RCT phase (Table 4).

### Match with Preferences and Algorithm Predictions

Only 39 SMHP users selected a plan choice during the dissemination phase by “starring” a plan on the website. Of these, 97% (38/39) of selected plans were good or moderate matches (good matches: 17/39, 44%; moderate matches: 21/39, 54%), and 22 matched one of the algorithm recommendations (22/39, 56%). The users in the RCT phase were more likely to have a match in the algorithm recommendations (*P*<.001) but not in the match score (*P*=.52; see Table 5), compared with the dissemination phase users.

**Table 4:** Importance ranking between the dissemination and randomized controlled trial (RCT) phases.

Question	Dissemination phase				RCT phase				Wilcoxon rank sum, |Z| approximation		*P* value
	n	Mean^a^ (SD)	Median (IQR^b^)	n		Mean^a^ (SD)	Median (IQR)				
Cost of health insurance premium	182	4.5 (0.9)	5.0 (1.0)	164		4.5 (0.9)	5.0 (1.0)	0.49		.62	
Cost of deductible	182	4.3 (1.0)	5.0 (1.0)	164		4.4 (0.9)	5.0 (1.0)	0.13		.89	
Cost of doctor visits	173	4.0 (1.1)	4.0 (2.0)	164		4.3 (1.0)	5.0 (1.0)	2.99		.003	
Cost of prescription pills or medicine	173	4.1 (1.1)	4.0 (2.0)	164		4.4 (1.0)	5.0 (1.0)	3.01		.003	
Choice of doctors, including some that are out-of-network	171	4.3 (1.1)	5.0 (1.0)	164		4.0 (1.2)	5.0 (2.0)	2.82		.005	
Cost of out-of-network care	173	3.2 (1.4)	3.0 (3.0)	164		4.0 (1.2)	5.0 (2.0)	5.45		<.001	
Fixed cost for tests or care	171	4.0 (1.1)	4.0 (2.0)	164		4.4 (0.9)	5.0 (1.0)	3.75		<.001	
Out-of-pocket maximum	173	4.3 (1.0)	5.0 (1.0)	164		4.6 (0.8)	5.0 (1.0)	2.49		.01	
Formulary	171	4.2 (1.2)	5.0 (2.0)	164		4.3 (1.1)	5.0 (1.0)	0.72		.47	

^a^Importance of features ranked from least to most important on a scale of 1 (not at all important) to 5 (very important).

^b^IQR: interquartile range.

**Table 5:** Match with preferences and algorithm predictions between the dissemination and randomized controlled trial (RCT) phases.

Variable		Dissemination phase, n (%)		RCT phase, n (%)		χ^2^		*P* value^a^	
**Choice match algorithm**						**12.5**		**<.001**	
	Yes		22 (56.4)		134 (82.7)				
	No		17 (43.6)		28 (17.3)				
**Match score**						**1.29**		**.52**	
	Good match		17 (43.6)		85 (52.5)				
	Moderate match		21 (53.9)		71 (43.8)				
	Poor match		1 (2.6)		6 (3.7)				

^a^Testing difference between dissemination and RCT phases.

## Discussion

### Principal Results

Dissemination of the Web-based SMHP health insurance decision support tool successfully reached a large number of users in the state of Missouri. Although this phase was successful in reaching many users, there were key lessons learned from this process. First, the dissemination tool users had a significantly higher income level compared to those in the RCT phase, suggesting that a higher income population may be more likely to seek information and online help when making a health insurance plan decision. Barriers that may hinder low income populations from seeking online insurance help could be limited time to access the internet, lack of interest seeking online information, lack of familiarity with tools or discomfort entering personal information online [27-29]. To better facilitate the dissemination of this tool to a larger target audience and increase its visibility, future work could incorporate the tool information into other assistant programs and websites. For example, since people are more likely to pay attention to personally relevant information, marketing the tailored tool on insurance enrollment websites could encourage users to engage with the tool. However, there are challenges associated with keeping the tool sustainable and updated, including the costs of advertising and dissemination as well as website maintenance.

In addition, our findings suggest that SMHP users were more selective in the information they reviewed and spent less time on all the sections in the dissemination phase compared with the RCT phase. In the dissemination phase, only a small number of SMHP users filled out their personal and family information and reviewed the final health insurance plan recommendations. This may indicate that people are reluctant to disclose personal health information online [30]. Prior studies have found that people’s perceived health information sensitivity influences their intention to disclose health information [31]. Online users chose to share private information when the perceived benefits outweighed the perceived risks [32,33]. For example, patients were willing to share electronic health data with their health care professionals, but were less inclined to permit secondary data use when there was a greater risk of confidentiality loss [34]. Collecting some private information is necessary for the implementation of an online tool that generates tailored health insurance recommendations, but the process of collecting personal information or the level of detail may be improved by promoting a model of trust and safety with information technology.

Low completion rate might also be linked to the current insurance status of SMHP users. For instance, SMHP users who were already enrolled in health insurance in the 2016 cycle may have completed the educational sections to learn more about health insurance. In this case, the *Eligibility* and *Your Plans* sections would not have been relevant to these users. In addition, users from outside the state of Missouri would not have benefited from the cost calculator, which was specific to Missouri plans; this may explain why some users did not complete the tool.

Furthermore, this study actively integrated evidence-based strategies for implementing change [25,26], built coalitions with local stakeholders and communities, and created a feedback loop in developing tool content to transform a research tool into a publicly available online tool. Damschroder et al [35] suggested that implementing a research tool in a particular setting requires the preservation of the essential and core elements of the tool as well as modification of adaptable elements based on the dissemination settings. The evidence-based strategies [25,26] were helpful for engaging stakeholders and community members to identify the core and adaptable elements in order to assess the likelihood of adoption of SMHP, along with potential barriers and facilitators to implementation. In addition, formatting visual and written context in a research study is different because research participants are more engaged in the study procedures and interventions, thus seeking feedback from stakeholders could potentially reduce user burden and make the tool robust across broader populations, especially for an audience with low health literacy.

However, even with these extensive strategies, many more users are available in the marketplace across the state, and additional work may need to be done in-person to promote the routine adoption of tools like SMHP. When a public health program proceeds to the dissemination phase, there are many confounding factors that might affect the utilization of the tool. For example, participants in the RCT phase had a better match score on health insurance plan recommendations compared to the users in the dissemination phase. Therefore, we cannot determine if any other factors impacted a user’s final choice in the dissemination phase.

### Limitations

One primary limitation of this study is that dissemination phase users were less likely to complete demographic questions. Sample sizes for demographic variables varied, as users often stopped before completing the entire tool. SMHP users were allowed to skip the sections that required them to enter their personal information, which is typical of dissemination in a real-world setting. In addition, we did not track SMHP users who used the tool under a different user ID as the user ID generated by the tool was not linked to an IP address or any other identifiable information. When we compared the users who began the *Eligibility* section of the tool to those who completed the *Eligibility* section and saw plans, fewer data were available from sections later in the tool. More SMHP users, therefore, reported age and county, which were asked earlier in the tool, but fewer reported income.

We assume that anyone who stayed on the website for one or more seconds was categorized as a SMHP user, indicating that those users did not open and then immediately close the tool. The reason for this assumption is based on page hits, as we were able to calculate time for anyone who clicked past the first page. This could lead to sampling bias given that we were only collecting data from SMHP users who may favor the SMHP tool. Additionally, because of skewed distributions in many instances, nonparametric tests were used for analyses.

### Conclusions

This study provides an example of the dissemination and implementation of an empirically tested Web-based decision aid tool for the general public. From this experience, we can conclude that disseminating this tool is feasible as it was able to attract potential users, educate them on basic health insurance terms, and make recommendations based on personal information and preference. In addition, this also serves as an example of a successful adoption of evidence-based recommendations for implementing change [25,26]. However, future research is needed to investigate the factors that impact online users’ information-seeking behaviors when using a public health information tool, as well as explore strategies that may engage low income populations.

## Abbreviations

ACA: Affordable Care Act
MEPS: Medical Expenditure Panel Survey
RCT: randomized controlled trial
SMHP: Show Me Health Plans
